# Clinical Observations in Private Practice

**Published:** 1851-01

**Authors:** E. B. Haskins

**Affiliations:** Clarksville, Tenn.


### WESTERN JOURNAL

OF

### MEDICINE AND SURGERY.

JANUARY, 1851.

### Art. I.—*Clinical Observations in Private Practice.* By Dr. E. B. Haskins, of Clarksville, Tenn.

"All is not then obscure or uncertain in medicine when the observations which guide us are exact."—*Louis.*

"To accuracy of observation appertain practice of the senses, an acquaintance with the terms of art, full attention to the subject, freedom from preconceived opinions, and a willingness to speak the truth."—*Schill.*

Observations made in private practice uudoubtedly possess as much value, *other things being equal*, as those made in public institutions. But as *other things* are *not* equal, it may not be amiss to notice, in this preliminary, some of the more salient points wherein the want of equality consists.

**1.** In private practice, and particulorly in the country, the physician has a different nurse for nearly every patient, and therefore, nurses of every degree of experience and grade of capacity, have to be relied upon for statements of what occurs in the *interim* of visits; hence, great circumspection is necessary on the part of the practitioner to obtain *trustworthy* information. Indeed many cases, otherwise of great interest, are rendered valueless on account of the ignorance of those in constant attendance. Besides, the *treatment* is often simplified to a fault, by reason of the danger of confiding too many provisions to the understanding and judgment of ignorant attendants.

2. In private practice, that freedom with the *person* of female patients, particularly of rank and fashion, in the various steps of an examination that accuracy demands, would often be regarded as indelicate and officious, except in very grave and alarming cases.

Napoleon on few occasions displayed more sagacity and wisdom than when he said to M. Dubois, on being informed of the critical condition of Maria Louisa in her accouchement, "go back to the Empress's chanber, and treat her as you would a baker's wife.

3. The distance from one patient to another is such as to require the physician so to encumber himself with instruments of diagnosis, therapeutical appliances, notebook, etc., as to force him often to forego the advantages derived therefrom.

4. The number of cases of any one disease, properly circumstanced for reliable record, is seldom sufficiently large, in private practice, to furnish data for the deduction of any law in pathology, or the demonstration of the efficacy of any method of treatment.

5. Post-mortem dissections can rarely be pursued to any very valuable extent in private practice. The subjects allowed the physician for examination are, at best, but few, and with these, the time allowed is generally too short to make other than hurried and imperfect examinations. The track of the alimentary canal and spinal marrow, can seldom be inspected at all under such circumstances.

On reviewing the difficulties and embarrassments here enumerated, that do not exist in hospital practice, it can hardly be wondered at that Dr. James Jackson, thirty years after seriously contemplating the plan of recording his observations, and providing himself with printed blanks for his cases, should make the public acknowledgment that "the difficulties attending the plan in private practice discouraged me [him] too soon." (See Preface to Louis on Bloodletting.)

It should not be overlooked, on the other hand, that observations made in private practice possess, in some particulars, a value that does not belong to those made in public institutions.

1. In hospitals, many patients enter from a distance, and, therefore, the predisposing, exciting, and modifying influences that have surrounded them cannot be accurately learned, either on account of ignorance, want of veracity, or the state of mind induced by disease. In private practice, on the contrary, family temperaments and predispositions can be ascertained; individual habits successfully inquired into; local and domestic causes examined—in a word, every thing may be learned that is necessary to a full history of each particular case.

2. In large metropolitan hospitals, there are *peculiar* influences, that so impress aud alter the natural aspect and course of disease, as to render observations made therein false types of disease generally, as well as fallacious and unsafe guides in general practice.

That the difficulties attending private practice may be so far overcome, by persevering industry, as to enable the practitioner to record his more interesting cases in such a manner as to benefit himself, will not be argued here, as no one will, for a moment, doubt such a proposition. It may not, however, be so readily granted, that their *publication*, amidst such a tide of hospital reports as flows through the medical periodical press at present, can, in any appreciable degree, advance the interest of medicine. Yet it will hardly be denied that whatever is of *individual*, may be of *general*, interest—that if it be useful to the individual practitioner to record his cases for the assistance of his own memory, and thus *acquire the largest amount of experience in the shortest possible time*, that, therefore, the same benefits will accrue to others who may avail themselves of such experience. The same rule holds in this case as that of hospital practice; with this difference, however, as regards their relative *importance*,—the private practitioner must content himself with an humbler, though no less responsible, share of clinical literature. Instead of aspiring to govern professional opinion by extensive monographs of special observations, he must content himself with furnishing such scattered materials as fall within the scope of his more diffuse labors; instead of essaying to erect a temple to his own fame, he must be satisfied with a *niche* in the great common temple, of which he can but act as an humble cobuilder. To this subordinate end, however, it can not be disguised, that industry, honesty, and capability are absolutely essential—without these requisites, clinical reports are mere trash, that only clog the literature, and embarrass the science of the profession, without the possibility of giving other than a brief notoriety to the author.

With this hasty exposition, then, of my sense of the comparative insignificance of my sphere of practical researches, as well as my responsibility to a rigid and jealous profession, I proceed to publish such observations as I have made at the bed-side, that present to my mind points of more or less practical interest.

OBSERVATION I.

*A case of Phlegmasia Dolens, treated locally with tinct. iodine—complete cure.*—Mrs. S——, aged 30 years, of leucophlegmatic temperament, was brought to bed of her sixth accouchement on the evening of the 4th of December 1847; birth easy and natural. About the sixth month of the next preceding gestation, she suffered a premature delivery. Near the same period of her last gestation, she was attacked with remittent fever, and had quite an irritable stomach. The remittent fever passed into an intermittent fever, which lasted a few weeks and terminated in health.

From the time of accouchement (4th) up to the 12th, the patient did well, except a slight headache on the 11th, for which a dose of castor-oil was ordered, (the bowels being confined). The oil was not taken until the 12th, when she felt quite well; sat up, and had appetite. The oil produced one copious dejection at night.

During night of the 12th, patient had slight shivering sensations, with wandering pains in the hypogastric region.

13th. *Morning.* Complains of chilliness; pain and soreness of bowels; no swelling; countenance distressed; tongue furred; no headache; some thirst; feeling of lassitude; pulse 100, and soft.

*Evening visit.* Pain has fixed itself suddenly in the left hip and groin, extending down left thigh, with inability to move the leg; no swelling; tongue foul and clammy; pulse 107, and soft. Calomel, grs. v, Dover's powder ℈ss, to be taken at night; a lotion of spt. camph., spt. ammon. and spt. terebinth, of each one part, laudanum two parts, to be applied, and limb enveloped in flannel.

14th. *Morning visit.* Slept but little during night; tongue foul and clammy; pulse 118 and soft; skin dry, and temperature natural; bowels not moved; limb swelling below the knee; greatest pain and tenderness in calf; anorexia; no lochia; milk suppressed. Two teaspoonfuls of sulph. mag. in half tumbler of water until bowels act; tinct. of iodine applied to limb from groin to ankle, with a soft shaving brush; lemonade; diet light. *Afternoon.* Pulse 130, full and soft; thirst; nausea; pain not so great, but confined to calf, swelling increases upwards, embracing the thigh; no action from bowels. Injections of salts and senna tea at short intervals until bowels act; lemonade for drink; light diet.

15th. *Morning.* Enemata produced free evacuation of bowels during night, after which ¼ gr. sul. morph. was given; patient slept but little; pulse 118, soft, with good volume; feeling of great debility; sick stomach; thirst; clammy tongue; urine sparce, and depositing lateritious sediment; leg less painful and tender, has not increased in size, but swelling extends from hip to ankle. Renewed application of tinct. of iodine; ℈ss. of nitrate of potass dissolved in a pint of lemonade, to be drank in course of the day; wine whey allowed for diet. *Evening.* Pulse 135, soft, and good volume; less nausea; thirst; tongue clammy; some headache; urine increased in quantity.—Lemonade.

16th. *Morning.* Pulse 118 and soft; rested well during night; less thirst; leg and thigh less painful, and swelling not increased; urine more abundant. Twenty drops of spts. nitre in ℥ij. of parsley-root tea every two hours; renewed application of tinct. iodine to limb. *Evening.* Pulse 112; less thirst; slight soreness of abdomen; urine sparce and muddy; leg not examined. Spts. nitre and tea continued; hot wilted tanzy to belly.

17th. Pulse 107, and soft; tongue moist; no thirst; limb less painful and slightly reduced in size; urine increased, and less muddy; two or three small dejections; abdomen less sore. Solution of cream tartar and spts. nitre every two hours; reapplication of tinct. of iodine. *Evening.* Pulse 112; tongue moist, and cleaning; leg painful, but not increased in size or tenderness; urine increased and less muddy. Cream tart. and spts. nitre continued—lemonade allowed.

18th. *Morning.* Slept but little last night on account of pain in swelled limb; otherwise feels well; pulse 105; some perspiration last night; tongue moist, and cleaning; appetite returning; countenance cheerful; bowels open; no tenderness of abdomen; urine natural in quantity and quality. Cream tartar and spts. nitre; reapplication of iodine tinct., with bottles, filled with hot water, to leg when painful. *Evening.* Pulse 112; burning sensation over the skin of affected limb; (owing to desquammation having taken place the iodine irritates the recently formed cuticle;) other symptoms as in morning.—Cream tartar and spts. nitre; application of cool cream to surface of leg and thigh.

19th. *Morning.* Pulse 108; feels well; slept well over night; swelling subsiding in upper part of limb, but slightly increased in ankle. Cream tartar and spts. nitre; wilted cabbage leaves* to limb. From this date the pulse rapidly reduced in frequency, and the limb, in equal ratio, in size from above downward, with other symptoms of returning health—leaving the foot and ankle edematous, (which soon yielded to moderate pressure by a flannel roller) and by the middle of January, 1848, patient was enabled to walk any where with ease, without assistance.

This case was a radical cure. The subject has borne another offspring without any return of symptoms The limb remains perfectly well, without swelling or stiffness. Another case treated on the same plan, by my suggestion, also yielded promptly to the treatment, and remains well.

* Few western practitioners but understand the soothing influence of cabbage leaves to irrirated surfaces. It seems to have taken the place of cerates, in the country, as an after dressing to blistered surfaces. Was the practice introduced by old women? or by the country surgeon who found it more convenient than to carry cerates with him? There is a popular belief that one side of the leaf draws or keeps the blister open, whilst the other side heals!

It will be remembered that Mr. John Davies, Surgeon to the General Infirmary at Hartford, has, in an elaborate paper,* insisted upon the efficacy of tinct. of iodine, topically applied in local inflammations generally, with or without fever. I have long been acquainted with its potency over local swellings and inflammations, unaccompanied by fever, but the above case would seem to entitle this remedy to some consideration as a *revulsive* in the acute phlegmasiæ. The tinct. I used was prepared according to the U. S. formula (Iodine ℥j; alcohol Oj.) diluted so as *just not to vesicate.* My notes do not inform me of the condition of the crural vein. I presume it was not found hard and corded; yet the omission was none the less an oversight, as the absence of important symptoms should always be noticed—*negative* symptoms being, often, as important as positive ones.

OBSERVATION II.

*A case of Puerperal Peritonitis—its accession simulating after pains—death—post mortem examination.—*

Feb. 18th, 1848.—Negress, aged about 28 years, the property of Dr. D., was delivered on the 14th inst. of her second child, which was vigorous and healthy at birth, but died on the 3d day from hemorrhage of the umbilicus. Accouchement by an old negress who delivered the patient sitting; parturition attended with no unusual circumstances, except the complaint of "cramping in the stomach" immediately after delivery, which was promptly relieved by a dose of laudanum. Dr. D. informs me that on the 15th and 16th, other anodynes were given for what he took to be after pains, being intermittent in their character.

On this morning (18th) finding the abdomen swollen and tender to pressure, Dr. D. opened a vein and drew

*This paper was republished in 1839, in Dunglison's Amer. Med. Lib.

about four ounces of blood, being detered from further depletion on account of approaching syncope.

Half past 8 o'clock, P. M.—Decubitus mostly dorsal with knees flexed, sometimes left lateral with flexed limbs, and belly thrust forward; pulse 145 and feeble; skin cool and dry, (and has been from beginning of disease;) tongue white and slightly coated; respiration thoracic and frequent; abdomen distended and tender to firm pressure; no tenderness in groins; vagina hot and puffy; cervix uteri, in situ, body of uterus not tender, nor of unusual dimensions; bowels confined; urine natural in quantity; lochia not suppressed; no sign of lactation; occasionally vomits up a greenish matter. Venesectionto f.℥xij; calomel ℈ss; opium grs. ij; a large wheat-bran and vinegar poultice to abdomen. Stimulants were likewise used; but patient progressively declined and expired on the 19th, at 7 o'clock, A. M.

*Post-mortem examination three hours after death.*—On opening the abdomen, a large quantity of clear straw colored serosity flowed out; a pus-colored coagulable lymph, in flakes of various sizes, was floating amongst the intestines; the peritoneal covering of the viscera, and abdominal parietes were white and shining; the bowels were slightly adherent wherever in contact; the ovaria were as large as a hen's egg, and were entirely enveloped in a firm pus-colored lymph—when scraped off, ovaria were red and vascular; broad ligaments covered with same sub stance; uterus appeared about the size of a small cocoanut and firm;—the veins of this organ, and its internal membrane were not examined, for want of the *liberty to do so;*—bowels were distended with gas.

There is nothing new in the pathological anatomy of this case—nor indeed in any other respect. It may be useful, however, as another proof of the deceptive invasion often, of the most fatal maladies; and should admonish us of the importance of the strictest attention to all abdominal and pelvic pains occurring in the puerperal woman. Dr. D. is an intelligent physician, and though he has retired from practice, is as little likely to make blunders of this sort, as hundreds of young practitioners in daily active practice.

The very exact likeness of the lymph found in the abdomen, to pus, has led me to inquire whether the pus found so often by the earlier laborers in this department of morbid anatomy may not have been coagulable lymph.

The last bleeding was certainly too late to do any thing but harm, and would not have been used but for the uncertainty of the time of attack. It was improper at any time, according to the rule laid down by Dr. Robert Lee, who says "we should not be deterred from employing the lancet because the pulse is small and contracted, provided it does not exceed 110 or 115 pulsations in the minute." In this case the pulsations were 145 in the minute.

(TO BE CONTINUED.)

